# Neuroprotection by aripiprazole against β-amyloid-induced toxicity by P-CK2α activation via inhibition of GSK-3β

**DOI:** 10.18632/oncotarget.22777

**Published:** 2017-11-30

**Authors:** So Youn Park, Hwa Kyoung Shin, Won Suk Lee, Sun Sik Bae, Koanhoi Kim, Ki Whan Hong, Chi Dae Kim

**Affiliations:** ^1^ Department of Pharmacology, School of Medicine, Pusan National University, Gyeongsangnam-do 50612, Republic of Korea; ^2^ Gene & Cell Therapy Research Center for Vessel-associated Diseases, Pusan National University, Gyeongsangnam-do 50612, Republic of Korea; ^3^ Department of Korean Medical Science, School of Korean Medicine, Pusan National University, Gyeongsangnam-do 50612, Republic of Korea

**Keywords:** aripiprazole, alzheimer’s disease, Wnt/β-catenin pathway, GSK-3β, CK2α

## Abstract

Psychosis is reported over 30% of patients with Alzheimer's disease (AD) in clinics. Aripiprazole is an atypical antipsychotic drug with partial agonist activity at the D_2_ dopamine and 5-HT_1A_ receptors with low side-effect profile. We identified aripiprazole is able to overcome the amyloid-β (Aβ)-evoked neurotoxicity and then increase the cell viability. This study elucidated the mechanism(s) by which aripiprazole ameliorates Aβ1-42-induced decreased neurite outgrowth and viability in neuronal cells. Pretreatment with aripiprazole increased Brain-derived neurotrophic factor (BDNF) mRNA and protein expressions in N2a cells. Additionally, phosphorylated casein kinase 2α at Y 255 (P-CK2α) was increased in time- and concentration-dependent manners. Furthermore, Aβ1-42-induced decreased BDNF and P-CK2α expression were increased over control level by aripiprazole. Subsequently, Aβ1-42-induced decreased levels of phosphorylated glycogen synthase-3β at Ser9 (P-GSK-3β) and nuclear P-β-catenin (Ser675) were elevated by aripiprazole, which were inhibited by K252A (inhibitor of BDNF receptor) and tetrabromocinnamic acid (TBCA, CK2 inhibitor), indicating that BDNF and P-CK2α activation are implicated in the aripiprazole effects. Expressions of cyclin D1 and insulin-like growth factor 2 (IGF2) mRNA were increased by aripiprazole; even in the presence of Aβ1-42, which was blocked by K252A and TBCA. In CK2α gene-silenced N2a cells, aripiprazole failed to increase P-GSK-3β and P-β-catenin expressions. Consequently, aripiprazole ameliorated Aβ1-42-induced attenuation of neurite elongation in HT22 cells, and this effect was blocked by both TBCA and imatinib. Decreased viability induced by Aβ1-42 was recovered by aripiprazole. These findings provide evidence supporting that aripiprazole can provide an effective therapeutic strategy against Aβ-induced neurotoxicity in AD-associated psychosis.

## INTRODUCTION

Alzheimer's disease (AD) is characterized by extracellular β-amyloid peptide (Aβ)-containing extracellular plaques and intracellular neurofibrillary tangles, accompanied by synaptic and neuronal dystrophy [1–3]. In addition, AD patients show several neuropsychiatric symptoms such as depression, agitation and psychosis (delusions, hallucinations), which have a negative impact on cognition [4].

Brain-derived neurotrophic factor (BDNF), the most abundant neurotrophin in the brain, has pivotal roles in synaptic plasticity and cognition [5]. Moreover, BDNF was demonstrated to inhibit GSK-3β activity through increased phosphorylation at serine 9 in cerebellar granule cells and human neuroblastoma SH-SY5Y cells [6]. The activation of the PI3K/Akt pathway by BDNF leads to inactivation of GSK-3β by phosphorylation at serine 9 [7]. Recently, aripiprazole was demonstrated to increase the BDNF level in the hippocampus of rats subjected to immobilization stress [8]. Furthermore, CK2 (casein kinase 2), a highly conserved tetrameric serine/threonine kinase, plays an essential role in stimulation of the β-catenin/Tcf-LEF pathway [9, 10]. In addition, many researchers have reported a reduction in pro-BDNF levels in brains of patients with AD [11, 12].

On the other hand, neuronal morphogenesis involves the formation and differentiation of neurites into axons and dendrites [13]. NGF-stimulated axonal elongation is occurred by activation of p75^NTR^ in cultured hippocampal neurons through inhibition of GSK-3β activity [14]. Reportedly, when β-catenin is stabilized, it translocates to nuclei, where it acts over Tcf/LEF sites and induces transcriptional activation [15]. β-catenin accumulation activates transcription of insulin-like growth factor (IGF)2 and cyclin D1 (a protein that promotes cell cycle entry), because the promoters of β-catenin have Tcf/LEF motifs [16]. Several studies have shown that antidepressants increase expression of IGF1 [7] and IGF2 [17]. In addition, IGF2 shows the increase in BDNF and IGF1 [18]. IGF2 mRNA level was reported to be declined in the frontal cortex of AD patients in early stages of neuropathology [19].

Aripiprazole, 7-{4-[4-(2,3-dichlorophenyl)-1-piperazinyl]-butyloxy}-3,4-dihydro-2(1H)-quinolinone, is an atypical antipsychotic drug with partial agonist activity at the D_2_ dopamine receptors; moreover, it has a potent partial agonist effect at 5-HT_1A_ receptors and an antagonist effect at 5-HT_2A_ receptors [20]. Aripiprazole has been licensed by the Food and Drug Administration (FDA) and European Medicine Agency (EMA) to treat schizophrenia in adults and adolescents [21], manic and mixed episodes with bipolar I disorder in children, adolescents, and adults [22], and major depression in adults [23, 24].

Schneider and Dagerman [25] have reported that prevalence of psychosis is estimated in patients with AD: range from 10 to 73% (median of 34%) within clinic populations. It has been known that declining of cognitive function in AD patients is associated with a high prevalence of psychotic symptoms [26] and behavioral disturbances [27]. De Deyn et al. [4, 28] have reported that in patients with psychosis associated with AD, aripiprazole-treatment showed significantly greater improvements in psychiatric rating scale compared to placebo and modest efficacy in the treatment of AD-related psychosis.

Given that aripiprazole is able to overcome the Aβ-evoked inactivation of Wnt/β-catenin by increasing the phosphorylated GSK-3β (Ser 9) through activation of CK2α, we hypothesized that aripiprazole might increase P-GSK-3β level and nuclear translocation of β-catenin with enhanced expression of cyclin D and IGF2 mRNA through increased BDNF production-linked activation of P-CK2α and thereby it can enhance neurite outgrowth.

## RESULTS

### Aripiprazole increases expression of BDNF mRNA and protein in N2a cells

**Figure 1:**
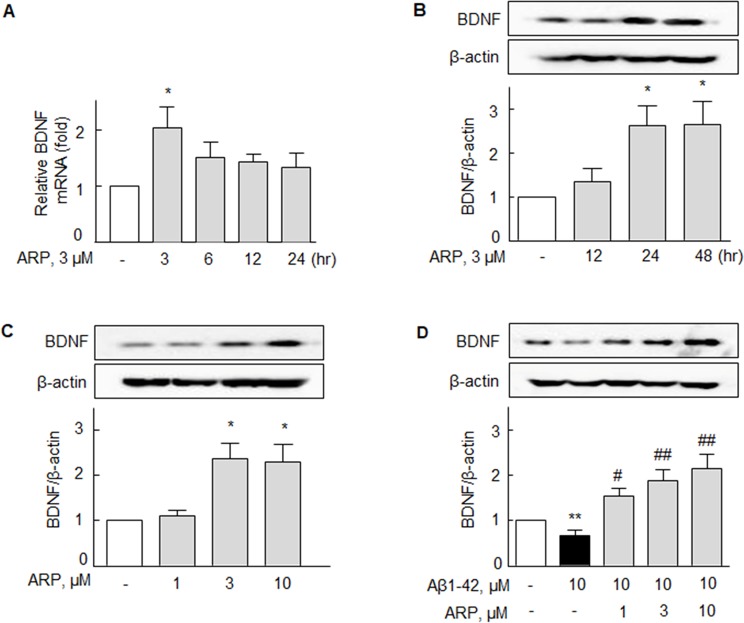
Aripiprazole-stimulated increase in BDNF mRNA and protein expression in N2a cells **(A, B)** Time-dependent increase in BDNF mRNA (0 - 3 hr) and protein (0 - 48 hr) expressions after treatment with aripiprazole (ARP, 3 μM). **(C)** Concentration (ARP, 1 - 10 μM)-dependent increase in BDNF protein expression (treatment for 24 hr). **(D)** Recovery of the Aβ1-42-induced decrease in BDNF by ARP. After pretreatment with ARP (1 - 10 μM) for 3 hr, cells were incubated with Aβ1-42 (10 μM) for 24 hr. Results are represented as mean ± SEM of duplicates each pooled 4 - 5 independent experiments. ^*^P < 0.05, ^**^P < 0.01 vs. None; ^#^*P* < 0.05, ^##^*P* < 0.01 vs. Aβ1-42 alone.

BDNF has been shown to have important roles in hippocampal synaptic plasticity [29] and memory function [30]. We assessed the increase in BDNF mRNA transcription and protein expression levels after treatment with aripiprazole in N2a cells. Following application of aripiprazole (3 μM) in N2a cells, the expression of BDNF mRNA was significantly elevated by 2.01 ± 0.38 fold (*P* < 0.05) at 3 hr, and subsequently declined at 6 - 24 hr after treatment (Figure 1A). Accordingly, the expression of BDNF protein after treatment with aripiprazole (3 μM) significantly increased in a time-dependent manner (0 - 48 hr), and reached a plateau at 24 - 48 hr (*P* < 0.05) (Figure 1B). The expression of BDNF protein at 24 hr also was elevated with increased concentration of aripiprazole (1 - 10 μM) (*P* < 0.05) (Figure 1C).

Some studies have reported a reduction in pro-BDNF levels in the brains of patients with AD [11, 12]. Cells that were previously exposed to Aβ1-42 (10 μM) for 3 hr were treated with aripiprazole (1 - 10 μM) for 24 hr. As shown in Figure 1D, Aβ1-42 exposure significantly decreased the expression of BDNF protein (up to 0.68 ± 0.11 fold, *P* < 0.01), and this decrease was prevented and rather elevated over the control by aripiprazole (3 and 10 μM) treatment to 1.88 ± 0.25 fold (*P* < 0.01) and 2.16 ± 0.30 fold (*P* < 0.01), respectively.

### Effect on P-CK2α (Y 255) and CK2α expressions

**Figure 2:**
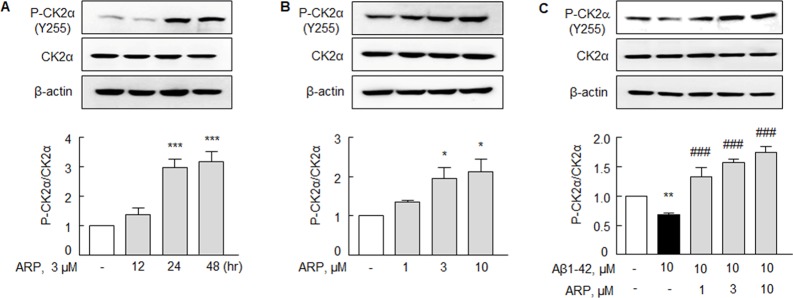
Aripiprazole stimulation of P-CK2α (Y 255) in N2a cells **(A, B)** Time (0 - 48 hr, by 3 μM ARP)- and concentration (1 - 10 μM ARP, treatment for 24 hr)-dependent increases in P-CK2α (Y 255) after exposure to aripiprazole (ARP). **(C)** Recovery of Aβ1-42-induced decreased levels of P-CK2α (Y 255) expression by ARP. After pretreatment with ARP (1 - 10 μM) for 3 hr, cells were incubated with Aβ1-42 (10 μM) for 24 hr. Results are represented as mean ± SEM of duplicates each pooled 4 independent experiments. ^*^P < 0.05, ^**^P < 0.01, ^***^P < 0.001 vs. none; ^###^*P* < 0.001 vs. Aβ1-42 alone.

Chao *et al.* [31] reported that BDNF increases protein kinase CK2 activity. Upon treating N2a cells with aripiprazole (3 μM), P-CK2α (Y 255) significantly increased in time (24 and 48 hr, *P* < 0.001)- and concentration-dependent (3 and 10 μM at 24 hr, *P* < 0.05) manners, but expression of CK2α was little changed (Figure 2A & 2B).

In our previous report, Aβ1-42 (10 μM) caused suppression of P-CK2α expression [32]. N2a cells were exposed to Aβ1-42 (10 μM) for 3 hr; subsequently, the cells were treated with aripiprazole (1 - 10 μM) for 24 hr. Under exposure to Aβ1-42, the expression of P-CK2α significantly decreased to 0.68 ± 0.03 fold (*P* < 0.01) (Figure 2C). This decrease was overwhelmingly surpassed over the control value by aripiprazole (1, 3, 10 μM): the expression of P-CK2α increased to 1.33 ± 0.03 fold (*P* < 0.001), 1.57 ± 0.06 fold (*P* < 0.001), and 1.74 ± 0.10 fold (*P* < 0.001), respectively.

### Effect on P-GSK-3β (Ser 9) expression

**Figure 3:**
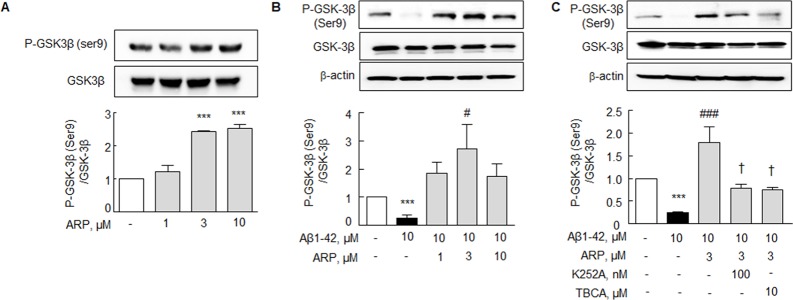
Aripiprazole stimulation of P-GSK-3β (Ser 9) in N2a cells **(A)** Aripiprazole (ARP) concentration-dependent increases in P-GSK-3β (Ser 9) level. Cells were incubated with ARP (1 -10 μM) for 24 hr. **(B)** Recovery by ARP of Aβ-induced decreased P-GSK3β (Ser 9) level. After pretreatment with ARP (1 - 10 μM) for 3 hr, cells were incubated with Aβ1-42 (10 μM) for 24 hr. **(C)** ARP-stimulated increased P-GSK-3β (Ser 9) level was blocked by K252A (BDNF receptor inhibitor) and TBCA (CK2 inhibitor). After pretreatment with ARP (3 μM) for 3 hr, cells were incubated with Aβ1-42 (10 μM) for 24 hr with or without K252A (100 nM) or TBCA (10 μM) for 30 min. Total GSK3β expression was little changed by ARP. Results are represented as mean ± SEM of duplicates each pooled 4 independent experiments. ^***^P < 0.001 vs. none; ^#^*P* < 0.05, ^###^*P* < 0.001 vs. Aβ1-42 alone. ^†^*P* < 0.05 vs. Aβ1-42 + ARP.

GSK-3β has been implicated in a wide range of disorders including neurodegenerative disorders, and the activity of GSK-3β is inhibited via phosphorylation at specific serine residues (serine 9 for GSK-3β) [33]. The levels of P-GSK-3β (Ser 9) was significantly decreased to 0.26 ± 0.12 fold (*P* < 0.001) by exposure to Aβ1-42 (10 μM) (Figure 3A). The Aβ1-42-induced decrease in P-GSK-3β (Ser 9) expression was rather elevated by aripiprazole (3 μM) to 1.79 ± 0.35 fold (*P* < 0.001). Interestingly, aripiprazole-stimulated increase in P-GSK-3β (Ser 9) expression was significantly decreased by K252A (a specific BDNF receptor inhibitor, 100 nM; *P* < 0.05) and TBCA (a CK2 inhibitor, 10 μM; *P* < 0.05) (Figure 3B). These results indicate that BDNF effect and CK2 activation are significantly implicated in aripiprazole-stimulated P-GSK-3β (Ser 9) expression.

### Effect on the increase in phosphorylated β-catenin (Ser 675) expression

**Figure 4:**
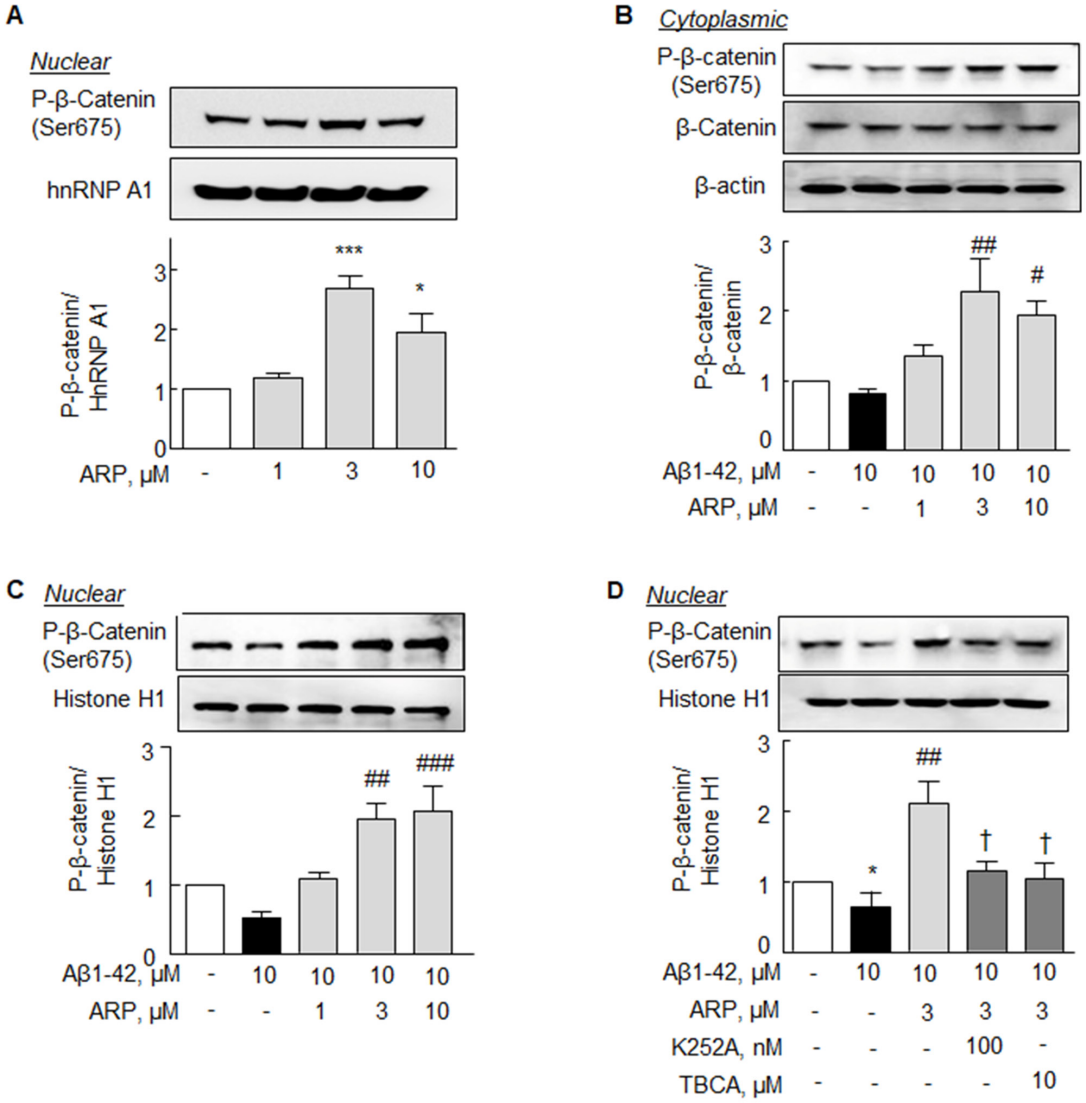
Increase in P-β-catenin at Ser 675 by aripiprazole treatment in the N2a cells **(A)** Aripiprazole (ARP)-stimulated concentration-dependent increases in nuclear P-β-catenin level. Cells were incubated with ARP (1 -10 μM) for 24 hr. ARP concentration-dependent increases in P-β-catenin (Ser 675) level under Aβ1-42 in the **(B)** cytoplasmic and **(C)** nuclear fractions of cells. After pretreatment with ARP (1 - 10 μM) for 3 hr, cells were incubated with Aβ1-42 (10 μM) for 24 hr. **(D)** Inhibition of ARP-stimulated increase in P-β-catenin (Ser 675) expressions by K252A and TBCA. After pretreatment with ARP (3 μM) for 3 hr, cells were incubated with Aβ1-42 (10 μM) for 24 hr with or without K252A (100 nM) or TBCA (10 μM) for 30 min. Results are represented as mean ± SEM of duplicates each pooled four independent experiments. ^*^P < 0.05 vs. none; ^#^*P* < 0.05, ^##^*P* < 0.01, ^###^*P* < 0.001 vs. Aβ1-42 alone. ^†^*P* < 0.05 vs. Aβ1-42 + ARP.

Ponce *et al*. [34] emphasized that the activation of CK2α enhances β-catenin transcriptional activity after increased nuclear import. The expression of P-β-catenin (Ser 675) in cytoplasm was marginally decreased by Aβ1-42 (10 μM). This P-β-catenin expression was significantly increased over the control level by aripiprazole (3 - 10 μM) (Figure 4A). In contrast, P-β-catenin (Ser 675) expression in the nuclear compartments was significantly decreased (0.54 ± 0.04 fold; *P* < 0.05) after exposure to Aβ1-42 (10 μM), and this decreased P-β-catenin was completely recovered by aripiprazole (3 and 10 μM) (Figure 4B). Furthermore, increase in nuclear P-β-catenin level by aripiprazole (3 μM) was markedly prevented by K252A (100 nM, *P* < 0.05) and TBCA (10 μM, *P* < 0.05) (Figure 4C). These results also indicate that aripiprazole-promoted nuclear translocation of P-β-catenin (Ser 675) is mediated via BDNF and CK2 activation.

### Increase in cyclin D1 and IGF2 in N2a cells

**Figure 5:**
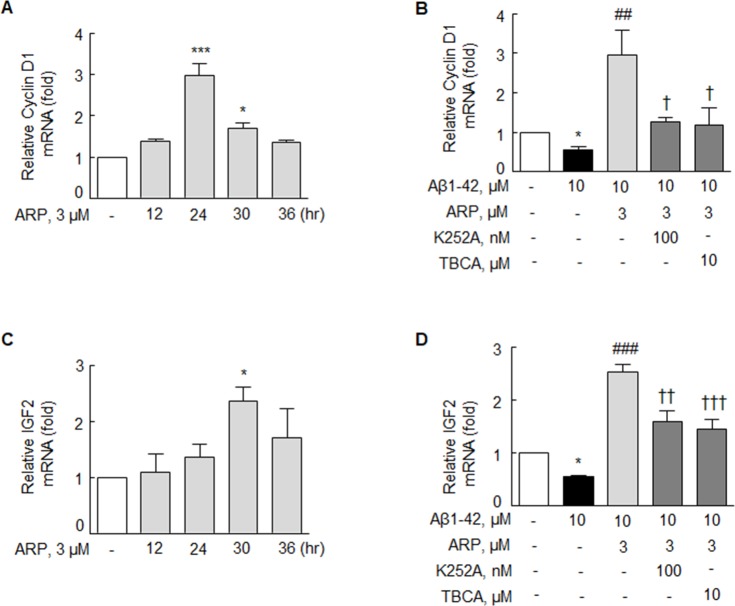
Effect of aripiprazole treatment on the expression of cyclin D1 and IGF2 mRNA in the N2a cells Time (0 - 36 hr) course effect of cyclin D1 mRNA **(A)** and IGF2 mRNA **(C)** expressions under aripiprazole (ARP) (3 μM). Inhibitory effects of K252A and TBCA on the ARP (3 μM)-upregulated cyclin D mRNA **(B)** and IGF2 mRNA **(D)** expressions. After pretreatment with ARP (3 μM) for 3 hr, cells were incubated with Aβ1-42 (10 μM) for 24 hr with or without K252A (100 nM) or TBCA (10 μM) for 30 min. Results are represented as mean ± SEM of duplicates each pooled 4 independent experiments. ^*^P < 0.05, ^***^P < 0.001 vs. none; ^##^*P* < 0.01, ^###^*P* < 0.001, vs. Aβ1-42 alone. ^†^*P* < 0.05, ^††^*P* < 0.01, ^†††^*P* < 0.001 vs. Aβ1-42 plus ARP (3 μM).

Cyclin D1 is an important regulator of G1/S phase cell cycle progression, and it is known to play an essential role in NGF-mediated differentiation [35]. We determined whether cyclin D1 is necessary to exert the proliferative effect of β-catenin signaling, since cyclin D1 has a role linked to the target genes of β-catenin. Following treatment with aripiprazole (3 μM), the expression of cyclin D1 mRNA was assessed over time (0 - 36 hr). As shown in Figure 5A, the mRNA of cyclin D1 was maximally induced at 24 hr to 2.97 ± 0.59 fold (*P* < 0.001) and thereafter declined. Further, cyclin D1 mRNA expression was significantly suppressed by Aβ1-42 (10 μM) to 0.56 ± 0.09 fold (*P* < 0.05). Upon treatment with aripiprazole (3 μM) in the presence of Aβ1-42 (10 μM), cyclin D1 mRNA expression was significantly increased to 2.94 ± 0.65 fold (*P* < 0.01), but the increase was completely attenuated by K252A (100 nM, *P* < 0.05) and TBCA (10 μM, *P* < 0.05) (Figure 5B).

To test the hypothesis that aripiprazole stimulates Tcf-LEF-mediated transcription of IGF2, the real-time PCR analyses were performed. Studies have been reported that IGF2 mRNA expression declines in the frontal cortex of AD patients at a relatively early stage of neuropathology [19], and that intrahippocampal injection of IGF2 in rat enhances memory function [36]. In this study, IGF2 mRNA expression was maximally increased at 30 hr after treatment with 3 aripiprazole (3 μM) to 2.36 ± 0.25 fold (*P* < 0.05) and then declined. The expression of IGF2 mRNA was suppressed by Aβ1-42 (10 μM) to 0.55 ± 0.05 fold (*P* < 0.05). Treatment with aripiprazole (3 μM) under Aβ1-42 (10 μM) significantly increased IGF2 mRNA expression to 2.53 ± 0.28 fold (*P* < 0.001), but this increase was blocked by K252A (100 nM, *P* < 0.01) and TBCA (10 μM, *P* < 0.001) (Figure 5C & 5D). These results strongly indicate that aripiprazole-stimulated cyclin D1 mRNA and IGF2 mRNA expressions are mediated via activation of BDNF and CK2.

### Effect of CK2α gene knockdown

**Figure 6:**
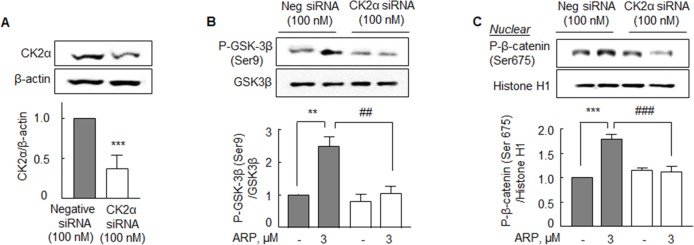
Analysis of CK2α-knockdown effects in the N2a cells **(A)** After CK2α gene silencing, CK2α protein expression was reduced to ∼63% of that in the negative control cells transfected with scrambled siRNA duplex. Aripiprazole (ARP) failed to elevate the levels of P-GSK3β (Ser 9) **(B)** or P-β-catenin (Ser 675) **(C)** in the N2a cells transfected with CK2α siRNA oligonucleotide (100 nM), as contrasted to the effects in the negative control cells transfected with scrambled siRNA duplex. Cells were incubated with ARP (3 μM) for 24 hr. Results are represented as mean ± SEM of duplicates each pooled 4 independent experiments. ^**^P < 0.01, ^***^P < 0.001 vs. negative siRNA; ^##^*P* < 0.01, ^###^*P* < 0.001 vs. ARP effect of negative siRNA group.

To confirm that the aripiprazole-stimulated elevation of P-GSK3β (Ser 9) and P-β-catenin (Ser 675) expressions are mediated via CK2α activation, N2a cells were transfected with CK2α siRNA. The transfection of CK2α siRNA resulted in reduction of CK2α to ∼63% of control level (Figure 6A). In negative control cells, aripiprazole (3 μM) significantly increased the expression of P-GSK3β (Ser 9) to 2.48 ± 0.50 fold (*P* < 0.01) and the expression of nuclear P-β-catenin (Ser 675) to 1.80 ± 0.10 fold (*P* < 0.001). However, aripiprazole failed to elevate the expressions of P-GSK3β (Ser 9) and nuclear P-β-catenin (Ser 675) in the N2a cells transfected with CK2α siRNA, as contrasted to the effects in the negative control cells transfected with scrambled siRNA duplex (Figure 6B & 6C). These results support the evidence that CK2α activation is crucially implicated in aripiprazole-stimulated P-GSK-3β (Ser 9) and P-β-catenin (Ser 675) expression.

### Effect on cell viability

**Figure 7:**
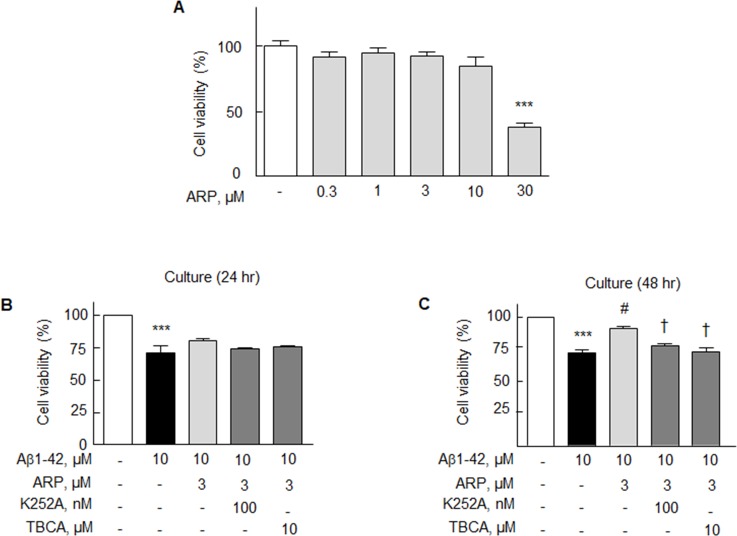
Effects of aripiprazole (ARP) on the cell viability of N2a cells **(A)** Cells were treated with various concentrations of aripiprazole (0.3 - 30 μM) for 24 hr in the culture after which the MTT assay was performed. **(B)** After pretreatment with ARP (3 μM) for 3 hr, cells were incubated with Aβ1-42 (10 μM) for 24 and 48 hr with or without K252A (100 nM) or TBCA (10 μM) for 30 min. Means ± SEM are expressed as percentages of none (N = 4). ^***^P < 0.001 vs. none; ^#^*P* < 0.05 vs. Aβ1-42 alone; ^†^*P* < 0.05 vs. Aβ1-42 + ARP.

N2a cells were treated with different concentrations of aripiprazole for 24 hr without Aβ1-42, after which the cell viability/cytotoxicity assay was performed. There was little change in cell viability up to 10 μM of aripiprazole, but 30 μM of aripiprazole caused significant decrease in viability to 38% (*P* < 0.001), indicating aripiprazole is relatively safe drug (Figure 7A). The cytotoxic effect of exogenously applied Aβ1-42 in N2a cells was assessed using a cell viability/cytotoxicity assay. Exposure of N2a cells to Aβ1-42 (10 μM) for 24 and 48 hr resulted in a significant decline in cell viability by 71.4 ± 3.0% (*P* < 0.001) and 71.8 ± 5.9% (*P* < 0.001), respectively. The decreased viability induced by Aβ1-42 was recovered by aripiprazole (3 μM) to marginally at 24 h, and significantly to 81.3 ± 3.2% (*P* < 0.05) at 48 h, which was blocked by K252A (100 nM, tropomyosin receptor kinase B (TrkB) receptor inhibitor) [43] and by TBCA (10 μM, CK2 inhibitor) [44] (Figure 7B & 7C). These results suggest BDNF and CK2 activation are involved in the aripiprazole-stimulated cell viability.

### Effect of aripiprazole on neurite elongation

**Figure 8:**
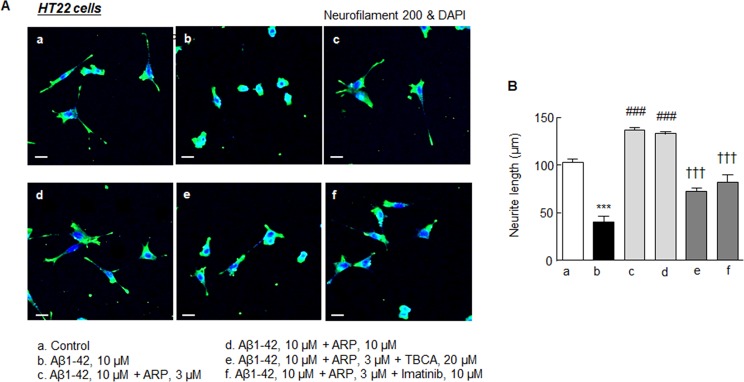
Effect of aripiprazole on the neurite elongation **(A)** Representative microscopic features. Recovery effect of aripiprazole (ARP, 3-10 μM) on the neurite elongation that had been inhibited by Aβ1-42 (10 μM) in HT22 cells in the absence and presence of TBCA (20 μM) or imatinib (10 μM, β-catenin inhibitor). Cells were cultured for 3 days. (Scale bar, 10 μm). **(B)** Results of quantitative analyses of neurite lengths (μm) are expressed as the mean ± SEM from six independent experiments. ^***^P < 0.001 vs. Control; ^###^*P* < 0.001 vs. Aβ1-42 alone; ^†††^*P* < 0.001 vs. Aβ1-42 + ARP.

Cultured HT22 cells, a stable murine cell line of hippocampal origin, expressing the BDNF receptor TrkB [37] were used to determine whether decreased neurite outgrowth induced by Aβ1-42 is recovered by aripiprazole, and whether this aripiprazole-recovered neurite elongation is, in turn, blocked by TBCA (a CK2 inhibitor) or imatinib (a β-catenin inhibitor). As shown in Figure 8, control neurite length (102.9 ± 3.7 μm) was significantly reduced to 40.7 ± 3.3 μm (*P* < 0.001) when cultured in medium with Aβ1-42 (10 μM). This decrease in neurite length was significantly recovered by aripiprazole (3 and 10 μM) to 136.5 ± 3.3 μm and 132.6 ± 2.8 μm, respectively (each *P* < 0.001). Further, aripiprazole-stimulated neurite elongation in the presence of Aβ1-42 was significantly blocked by TBCA (20 μM) and imatinib (10 μM) [38]. These results indicate that activation of CK2α and β-catenin is importantly involved in aripiprazole-stimulated neurite outgrowth of HT22 cells.

## DISCUSSION

The results of this study demonstrates that aripiprazole enhances neurite outgrowth and cell viability in the presence of Aβ1-42 by enhancing BDNF production and suppressing Aβ-induced GSK-3β activation, and thereby promoting nuclear translocation of P-β-catenin and increasing expression of cyclin D1 and IGF2 in the nucleus via enhancement of P-CK2α activation.

BDNF, a neurotrophin family member, has important roles in hippocampal synaptic plasticity [29] and memory function [30]. Some researchers have reported a reduction in pro-BDNF levels in AD brains [11, 12]. Wnt signal activation has a role in rescuing neurons from degeneration and improves animal behavioral impairments induced by β-amyloid fibril [39, 40]. We observed the increase in BDNF mRNA transcription and protein expression after treatment with aripiprazole in N2a cells. Even though application of Aβ1-42 significantly decreased the expression of BDNF, the decreased BDNF level overwhelmingly surpassed the control levels by treatment with aripiprazole. BDNF has critical functions in promoting survival and differentiation of neural stem cells via activation of Wnt/β-catenin signaling molecules [41].

Given that BDNF increases CK2 activity, we assessed the increase of P-CK2α (Y 255) expression after aripiprazole treatment. The expression of P-CK2α was significantly increased time- and concentration-dependently in N2a cells by aripiprazole without changing total CK2α expression. As Lee et al. [32] have indicated, P-CK2α expression was significantly decreased in response to Aβ1-42 in this study: Aβ1-42-induced decreased P-CK2α level was significantly recovered over the control value (by 1.3 ∼ 1.7 fold) under pretreatment with aripiprazole. It is widely known that GSK-3β is inhibited via phosphorylation at specific serine residues (*e.g*., serine 9 for GSK-3β) [33], and accumulation of active GSK-3β has been implicated in neurofibrillary degeneration in AD [42]. As predicted, the level of P-GSK-3β (Ser 9) was significantly decreased to ∼ 0.24 fold (*P* < 0.001) by Aβ1-42, but following treatment with aripiprazole, the decreased P-GSK-3β level was elevated. The increased P-GSK-3β levels were significantly blocked by K252A (BDNF receptor inhibitor) [43] and by TBCA (a CK2 inhibitor) [44]: these findings indicate that BDNF and CK2 activation are involved in aripiprazole-stimulated P-GSK-3β levels. CK2 is also implicated in Wnt signaling, where it acts as a positive regulator by phosphorylation of β-catenin, thereby leading to resistance to degradation by the proteasome and increased co-transcriptional activity [45]. As CK2α inhibits GSK-3β by phosphorylation at Ser 9, it was hypothesized that aripiprazole must stabilize and translocate β-catenin to the nucleus. Balaramana et al. [46] suggested that Wnt/β-catenin activity was notably low in AD patients’ brain. Consistent with this report, upon exposure of N2a cells to Aβ1-42, the level of P-β-catenin (Ser 675) was significantly decreased in the nuclear compartments. Interestingly, decreased nuclear P-β-catenin level was significantly elevated by aripiprazole, and these increases were completely blocked by K252A and TBCA. These results strongly suggest that aripiprazole-promoted nuclear translocation of P-β-catenin implies activation of BDNF and CK2α. These results support those reported by Sinha et al. [17] showing that inhibition of GSK3β activation is important for maintaining viability and activating the Wnt pathway.

Previous reports have shown that β-catenin activates the transcription of cyclin D1 (indicative of a promitogenic cell response) through TCF-binding sites within the promoter, which has a direct effect on cell proliferation [16] and through IGF2, a potent proliferative signaling protein [17]. In the present study, aripiprazole significantly increased the expressions of cyclin D1 and IGF2 mRNA, which had been suppressed by Aβ1-42. These increased mRNA expressions were blocked by both K252A and TBCA, indicating that aripiprazole-stimulated expression of cyclin D1 and IGF2 mRNA implies activation of BDNF and CK2α.

The postulation that aripiprazole-stimulated elevations of P-GSK-3β (Ser 9) and P-β-catenin (Ser 675) expressions are mediated via CK2α activation was further confirmed using N2a cells transfected with CK2α siRNA. After silencing the CK2α gene, the expressions of P-GSK-3β and P-β-catenin were not induced by aripiprazole, whereas negative control cells were obviously responsive to aripiprazole. It has been demonstrated that activation of CK2 by NGF enhances neurite extension in PC12 cells [47]. In addition, depletion of CK2 by antisense oligonucleotide has been reported to inhibit neuritogenesis in neuroblastoma cells, indicative of the importance of CK2α activation in neurite elongation [14]. In the present study, HT22 cells, mouse hippocampal neuronal cell line, were used instead of N2a cell, because HT22 cells phenotypically resemble neuronal precursor cells expressing BDNF receptor TrkB, and lack functional ionotropic glutamate receptors [37, 48], thus it was possible to exclude excitotoxicity as a cause for neurite outgrowth damage by glutamate other than Aβ1-42.

The Aβ1-42-induced decrease in neurite length in HT22 cells was prevented by aripiprazole, and the recovered neurite elongation was blocked by TBCA (CK2 inhibitor) and imatinib (β-catenin inhibitor), these findings indicating that activation of CK2α and β-catenin is importantly implicated in aripiprazole-stimulated neurite outgrowth in HT22 cells. Considering that IGF2 increases hippocampal levels of NGF, BDNF, and NT3 to varying degrees in animal model AD [18], it is suggested that BDNF is importantly involved in the aripiprazole-stimulated neurite outgrowth in support of critical roles in the function and survival of neurons.

It is known that aripiprazole's mechanism of action is pharmacologically ascribed to a combination of partial agonistic activity at D_2_ and 5-HT_1A_ receptors and antagonistic activity at 5-HT_2A_ receptors. Shioda et al. [49] have proposed that nuclear calcium/calmodulin-dependent protein kinase II (CaMKII) functions in transcriptional activation in the neurotrophin BDNF through the phosphorylation of diverse nuclear proteins, including CREB. However, it remains undefined as to the mechanism by which aripiprazole stimulates BDNF synthesis is related to D_2_ dopamine receptors, and/or to agonistic activity of 5-HT_1A_ receptors or antagonistic activity of 5-HT_2A_ receptors. This goes beyond the scope of the current study.

Considering these results are related to pharmacological inhibition and genetic blockade of CK2α, it is concluded that the activation of BDNF-coupled P-CK2α (Y 255) by aripiprazole stimulates expression of cyclin D1 and IGF2 mRNA through mediation of P-GSK-3β (Ser 9) and nuclear P-β-catenin (Ser 657), thereby contributing to neurite outgrowth and cell viability, even in the presence of Aβ1-42.

## MATERIALS AND METHODS

### Reagents and antibodies

Aripiprazole, 7-{4-[4-(2,3-dichlorophenyl)-1-piperazinyl]-butyloxy}-3,4-dihydro-2(1H)-quinolinone, was donated by Otsuka Pharmaceutical (Tokyo, Japan). Antibodies for anti-BDNF (Cat. No. sc546), anti-CK2α (Cat. No. sc12738) and anti-hnRNP A1 (Cat. No. sc32301) were from Santa Cruz Biotechnology Inc. (Santa Cruz, CA). Anti-β-catenin (Cat. No. 9562), anti-β-catenin phosphorylated at Ser 673 (Cat. No. 9567), anti-GSK3β (Cat. No. 9832), and anti-GSK3β phosphorylated at Ser 9 (Cat. No. 9339) were obtained from Cell Signaling (Danvers, MA). Anti-phospho-CK2α was from Invitrogen (Cat. No. PA5-40226, San Diego, CA). β-actin antibody was purchased from TRANSBIONOVO (Cat. No. HC201, Beijing, China). Aβ1-42 peptide was purchased from AnaSpec (Fremont, CA). TBCA [(E)-3(2,3,4,5-tetrabromophenyl) acrylic acid] was from EMD Chemicals (Gibbstown, NJ). K252A was from Calbiochem (San Diego, CA) and imatinib was from Toronto Research Chemicals (Toronto, Canada).

### Cell culture

The N2a, wild-type cells, a mouse neuroblastoma cell line, were provided by Dr. Takeshi Iwatsubo (Department of Neuropathology and Neuroscience, Graduate School of Pharmaceutical Sciences, The University of Tokyo) and cultured in DMEM supplemented with 10% FBS. N2a cells (neuroblastoma cell line) has been described to produce low levels of tyrosine hydroxylase and dopamine, and differentiate into dopamine neurons. Both tyrosine hydroxylase and dopamine levels were significantly enhanced by cAMP responsive element binding protein (CREB) [50]. HT22 cells, a murine hippocampal cell line, were donated by Dr. H.T. Chung (Ulsan University, Ulsan, Korea) and cultured in DMEM supplemented with 10% FBS.

### Western blot analysis

Proteins were loaded into 10% SDS-polyacrylamide electrophoresis gels, electrophoresed, and transferred to nitrocellulose membranes (Amersham Biosciences, Piscataway, NJ) that were incubated with anti-BDNF, anti-CK2α, anti-phosphorylated CK2α, anti-β-catenin, anti-β-catenin phosphorylated at Ser 673, anti-GSK3β, and anti-GSK3β phosphorylated at Ser 9 antibodies. Immunoblots were visualized by chemiluminescence using the Supersignal West Dura Extended Duration Substrate Kit (Pierce Chemical, Rockford, IL). Signals from bands were quantified by using a GS-710 calibrated imaging densitometer (Bio-Rad, Hercules, CA).

### RT-qPCR analysis

Total RNA was isolated from cells by using TRIzol reagent (Invitrogen). cDNA was synthesized from 1 μg of total RNA. Gene expressions were measured by performing real-time PCR using a LightCycler 96 system (Roche Molecular Biochemicals, Mannheim, Germany) equipped with LightCycler DNA Master SYBR Green I (Roche Molecular Biochemicals). PCR was performed under the following conditions: 95°C for 10 min followed by 50 amplification cycles of (95°C for 10 s, 50°C for 10 s, and 72°C for 10 s). Primers sequences are detailed in Table 1. Quantification was performed by using LightCycler 96 Software (Roche Molecular Biochemicals).

**Table 1:** Primer sequences for RT-qPCR

	Sense primer	Antisense primer	Accession number
**BDNF**	GGAAATCTCCTGAGCCGAGC	AGCTTTCTCAACGCCTGTCA	NM_007540.4
**Cyclin D1**	GAGCTGCTGCAAATGGAACTG	GGAGGGTGGGTTGGAAATGAA	NM_007631.2
**IGF2**	CCCCAGCCCTAAGATACCCT	GGGTATGCAAACCGAACAGC	NM_010514.3
**Actin**	GGAAATCGTGCGTGACATCAA	GAAGGCTGGAAAAGAGCCTCA	NM_007393.5

### Small interfering RNA preparation and transfection

CK2α small interfering (si)RNA oligonucleotide (GenBank accession No. NM_009974.2) was synthesized by Bioneer (Daejeon, Korea). siRNA molecules were transfected into cells by using X-tremeGENE siRNA transfection reagent (Roche, Indianapolis, IN) according to the manufacturer's instructions.

### Quantification of neurite elongation

To observe neurite elongation, HT22 cells, a stable murine hippocampal cell line, were plated at a density of 1,000 cells per cm^2^ on sterile, coated, 18 × 18-mm cover slips in a six-well culture plate. HT22 cells were incubated with Aβ1-42 (10 μM) alone or with aripiprazole (10 μM) in the absence and presence of inhibitors for 5 days. For the morphometric analysis, cells were fixed in 4% paraformaldehyde for 15 min at room temperature and then incubated with SMI-312 antibody (Cat. No. SMI312R, Covance, Princeton, NJ) for 1 hr. After a series of washes with PBS, secondary antibody conjugated to Alexa Fluor 488 (Invitrogen, Carlsbad, CA) was applied for 1 hr. All fluorescent images were magnified at ×400 by using an Axiovert 200 fluorescence microscope (Zeiss, Oberkochen, Germany). The length of the main neurite of each cell was measured in five independent experiments that were performed in duplicate.

### Cell viability

Cell viability was evaluated using the Cyto XTM cell viability assay kit (LPS solution, Daejeon, Korea). For viability assay, cells were treated with 10 % Cyto XTM per well, and again incubated at 37°C in a 5% CO2 incubator for 3 hr. Sample absorbance was determined at 450 nm using an ELISA (BioTek Inc., Winooski, VT).

### Statistical analysis

Results are expressed as mean ± SEM values. The significances of results were determined by performing one-way analysis of variance (ANOVA) followed by Tukey's Multiple Comparison Test. The Student's *t*-test was used to determine the significances of treatment effects. *P* values of < 0.05 were considered significant.

## Abbreviations

AD: Alzheimer's disease
Aβ: β-amyloid peptide
BDNF: brain-derived neurotrophic factor
CK2: casein kinase 2
IGF2: insulin-like growth factor 2
NGF: nerve growth factor
TCF/LEF: T-cell-specific transcription factor/lymphoid enhancer-binding factor-1.
